# UK Medical Cannabis Registry: Assessment of clinical outcomes in patients with insomnia

**DOI:** 10.1002/brb3.3410

**Published:** 2024-02-09

**Authors:** Kavyesh Vivek, Zekiye Karagozlu, Simon Erridge, Carl Holvey, Ross Coomber, James J. Rucker, Mark W. Weatherall, Mikael H Sodergren

**Affiliations:** ^1^ Imperial College Medical Cannabis Research Group, Department of Surgery and Cancer Imperial College London London UK; ^2^ Department of Medicine Curaleaf Clinic London UK; ^3^ Department of Trauma and Orthopaedics St. George's Hospital NHS Trust London UK; ^4^ Department of Psychological Medicine Kings College London London UK; ^5^ National and Specialist Tertiary Referrals Affective Disorders Service South London & Maudsley NHS Foundation Trust London UK; ^6^ Department of Neurology Buckinghamshire Healthcare NHS Trust Amersham UK

**Keywords:** benzodiazepines, cannabis‐based medicinal products, insomnia, sleep quality

## Abstract

**Introduction:**

The primary aim of this study was to assess changes in sleep‐specific health‐related quality of life (HRQoL) for those prescribed cannabis‐based medicinal products (CBMPs) for insomnia.

**Methods:**

A case series of UK patients with insomnia was analyzed. Primary outcomes were changes in the Single‐Item Sleep‐Quality Scale (SQS), Generalized Anxiety Disorder‐7 (GAD‐7), and EQ‐5D‐5L at up to 6 months from baseline. Statistical significance was identified as a *p* value < .050.

**Results:**

61 patients were included in the analysis. There was an improvement in the SQS from baseline at 1, 3, and 6 months (*p* < .001). There were also improvements in the EQ‐5D‐5L Index value and GAD‐7 at 1, 3, and 6 months (*p* < .050). There were 28 (45.9%) adverse events recorded by 8 patients (13.1%). There were no life‐threatening/disabling adverse events.

**Conclusion:**

Patients with insomnia experienced an improvement in sleep quality following the initiation of CBMPs in this medium‐term analysis. Fewer than 15% of participants reported one or more adverse events. However, due to the limitations of the study design, further investigation is required before definitive conclusions can be drawn on the efficacy of CBMPs in treating insomnia.

## Graphical abstract

This observational study evaluates the outcomes of patients with insomnia enrolled on the UK Medical Cannabis Registry. Changes in sleep quality were observed at up to 6 months, with 13.1% of patients reporting an adverse event.

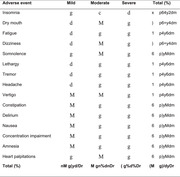

## 1 INTRODUCTION

Insomnia disorder is defined as persistent dissatisfaction with sleep quality or quantity for a minimum of three nights per week, despite adequate opportunity for sleep, lasting more than three months, causing significant distress or functional impairment (APA, 2013). Additionally, the impairment is not better explained by another sleep‐wake disorder, coexisting health condition, or drug effects (Sutton, 2021). The most common disturbances are sleep initiation, sleep maintenance, or early‐morning wakening (APA, 2013). Approximately 10% of adults are estimated to meet the insomnia disorder diagnostic criteria (Morin et al., 2011; Ohayon, 2002; Ohayon & Reynolds, 2009; Roth et al., 2011). Insomnia has a broad effect on health‐related quality of life (HRQoL) affecting biopsychosocial function, in addition to self‐perceived energy levels (Kyle et al., 2010; Lucena et al., 2020).

The exact pathophysiology of insomnia disorder is unknown. However, it is a heterogeneous condition which is best described using a biopsychosocial model of disease influenced by a broad range of predisposing (e.g., genetic vulnerability), precipitating (e.g., acute stressor such as relationship breakdown or bereavement), and perpetuating factors (e.g., chronic ill health, poor response to treatments) (Bastien, 2011; Becker et al., 2015; Bonnet et al., 2014; Hauri, 1991). Psychological interventions including sleep restriction, stimulus control, relaxation training, and cognitive‐behavioral therapy are all supported by Oxford Centre for Evidence‐Based Medicine level 1 evidence of efficacy (Kryger et al., 2010). Several pharmacological therapies are also utilized in the management of insomnia disorder. One of the most frequently prescribed classes of medications for insomnia are benzodiazepines, allosteric modulators of γ‐aminobutyric acid A (GABA_A_) receptors, which lead to broad inhibition of the central nervous system (Downing et al., 2005). It has been estimated that 300,000 adults in England have received a prescription for benzodiazepines for one year or more, above the recommended 4‐week use of these medications (Davies et al., 2017). Z‐drugs (imidazopyridines, pyrazolopyrimidines, and cyclopyrrolones) are also licensed for use in insomnia and act similarly through allosteric modulation of GABA_A_ (Krystal, 2009). However, there is a significant concern for the risk of abuse with these classes of medication (Krystal, 2009). Melatonin, which is involved in the regulation of the sleep‐wake cycle and promotion of sleep (Karasek & Winczyk, 1997), is approved for the treatment of insomnia in patients over 55 years of age by the European Medicines Agency (Clay et al., 2013). Melatonin has demonstrated a well‐tolerated safety profile and effects in improving sleep latency; however, it is not yet clear if these effects are clinically significant (Low et al., 2020).

Despite the availability of pharmacological therapies for insomnia, the rates of remission for chronic insomnia are poor and there are concerns regarding the risks of dependency with long‐term prescribing of benzodiazepines and Z‐drugs, demonstrating a need to identify emerging therapies (Davies et al., 2017; Janson et al., 2001; Krystal, 2009). The endocannabinoid system (ECS) has been suggested as a target for such therapies due to the evidence of its role in regulating the sleep‐wake cycle.

The ECS is an endogenous system of ligands, receptors, and enzymes, which are ubiquitous throughout the central nervous system, but also peripheral tissues, and plays a regulatory role in neurotransmission (Cristino et al., 2020). The primary receptors of the ECS are cannabinoid receptors—cannabinoid receptor 1 (CB1) and cannabinoid receptor 2 (CB2). CB1 is predominantly located in the central nervous system, where anandamide, an endogenous CB1 agonist, has demonstrated in preclinical studies promotive effects on rapid eye movement sleep, as well as wakefulness (Cristino et al., 2020; Murillo‐Rodríguez et al., 2001).

Cannabis‐based medicinal products (CBMPs) are derived from the cannabis plant, which contains over 140 phytocannabinoids, which interact with the ECS (Sampson, 2021). Preclinical evidence of CBMPs as a therapeutic agent for insomnia is promising (Mackie, 2008; Martinotti et al., 2012, 2011; Murillo‐Rodríguez, 2008; Ricci et al., 2021). The most abundant active pharmaceutical components of CBMPs are (−)‐trans‐Δ9‐tetrahydrocannabinol (Δ9‐THC) and cannabidiol (CBD). Δ9‐THC is a partial agonist of CB1 and CB2, while CBD is a negative allosteric modulator of CB1 (Laprairie et al., 2015; Paronis et al., 2012). However, the primary mechanism of action for CBD is through the inhibition of catalytic breakdown of anandamide an endogenous CB1 agonist (Elmes et al., 2015).

Previous research on CBMPs in insomnia has been limited by heterogeneity in studied medications and clinical populations, short follow‐up times, confounding by comorbid conditions, and small sample sizes (Kuhathasan et al., 2019; Kwak et al., 2020; Sznitman et al., 2020). The effects of CBMPs on sleep in the setting of treating other conditions are promising for secondary effects on sleep outcomes (Weinkle et al., 2019). This is particularly true in psychiatric conditions, such as post‐traumatic stress disorder, in which poor sleep quality is a core feature (Orsolini et al., 2019). Observational studies conducted utilizing data from the UK Medical Cannabis Registry (UKMCR), and other international data sets, have found that CBMPs are associated with improvements in the quality of sleep across all conditions (Olsson et al., 2023; Rifkin‐Zybutz et al., 2023; Sznitman et al., 2020; Tait, Erridge, Holvey, et al., 2023; Vigil et al., 2018). While clinically significant improvements in disease‐specific outcomes have been demonstrated in evaluations of those with generalized anxiety disorder and chronic pain, there has yet to be a bespoke analysis of individuals treated primarily for insomnia (Rifkin‐Zybutz et al., 2023; Tait, Erridge, Holvey, et al., 2023). Moreover, a recent study of a pharmaceutical preparation (ZTL‐101) containing Δ9‐THC (20 mg/mL), CBD (1 mg/mL), and cannabinol (2 mg/mL) found that it was well tolerated and improved sleep quality in patients with chronic insomnia. Furthermore, there were no serious adverse effects (AEs) observed. The results observed over the 2‐week dosing period were promising and support the further investigation of CBMPs for insomnia treatment across larger patient cohorts (Walsh et al., 2021). Despite initial promising findings, there is limited evidence of the efficacy and safety of long‐term prescribing. Consequently, no recommendations exist at present supporting the routine use of CBMPs in the treatment of insomnia on a population basis. There are concerns that long‐term consumption of Δ9‐THC can lead to tolerance to its effects on sleep (Babson et al., 2017). Moreover, while illicitly‐sourced cannabis is frequently used by self‐medicating individuals with insomnia, observational data suggest an association with adverse mental health outcomes, including psychosis (Couch et al., 2019; Martinotti et al., 2012, 2011; Ricci et al., 2021). However, observational data suggests that its incidence among medical cannabis patients is low (Elser et al., 2023; Zongo et al., 2022). In the absence of randomized controlled trials (RCTs), real‐world evidence in the form of patient registries can help inform clinical practice and future research directions. The primary aim of this study is to analyze changes in sleep‐specific patient‐reported outcomes in patients enrolled on the UKMCR who have been prescribed CBMPs for insomnia. The secondary aims are to examine if there is a change in general HRQoL following the initiation of CBMPs and assess safety.

## 2 MATERIALS AND METHODS

### 2.1 Study design

Data were extracted on February 15, 2022 from the prospectively designed UKMCR of patients treated with CBMPs for insomnia. The UKMCR has received approval from the South West–Central Bristol Research Ethics Committee (Ref: 22/SW/0145). All participants were enrolled consecutively and provided written informed consent.

### 2.2 Setting and participants

The UKMCR is a patient registry, established in December 2019, which longitudinally captures pseudonymized data of patients prescribed CBMPs from clinical encounters in the United Kingdom and Channel Islands and is privately owned by Curaleaf Clinic (Tait, Erridge, & Sodergren, 2023). Data are collected via a bespoke electronic reporting environment, which study participants have previously indicated as being easy to use for reporting patient‐reported outcome measures (PROMs) (91.6%), medications (85.0%), and adverse events (73.1%) (Tait, Erridge, & Sodergren, 2023).

All prescriptions for CBMPs were made in line with UK regulations, whereby individuals had to previously demonstrate a prior diagnosis of insomnia that had failed to improve following administration of more than two licensed treatments (Case, 2020). Moreover, assessment for suitability was conducted by an attending‐level neurologist, supported by a multidisciplinary team of attending‐level physicians from other relevant specialties (e.g., psychiatry) (Case, 2020).

During the initial clinical encounter, demographic data including age, gender, occupation, and previous medical history were recorded. The body mass index (BMI) was calculated for each participant according to reported height and weight. Primary, secondary, and tertiary indications for treatment with CBMPs were recorded. The Charlson Comorbidity Index, a tool which predicts the short‐ and long‐term mortality of an individual, was calculated for each participant in line with other disease registries (Brusselaers & Lagergren, 2017; Charlson et al., 1987). In addition, the incidence of hypertension, depression or anxiety, arthritis, epilepsy, venous thrombus embolus (VTE), and endocrine dysfunction were recorded. Drug and alcohol data, smoking status, and past cannabis use were recorded as described by our group previously (Ergisi et al., 2022; Harris et al., 2022).

The inclusion criteria for the present study were as follows: individuals aged greater than 18 years old with insomnia, initiated on CBMP therapy. Individuals with other indications for treatment with CBMPs were included if the primary reason for treatment was for insomnia disorder. Participants were excluded if the data of initiating medical cannabis were less than 1 month before the date of extraction from the UKMCR (February 15, 2022). Those who had not completed PROMs at baseline were also excluded from the analysis.

### 2.3 Exposure of interest

Data regarding CBMP prescriptions were recorded throughout directly from each written prescription, including manufacturing company, formulation, route of administration, Δ9‐THC and CBD doses, and strain. All CBMP prescriptions were manufactured according to Good Manufacturing Practice (GMP) criteria (Case, 2020). The dosages of CBMPs were determined by the multiplication of the concentration (mg/ml or mg/g) and the daily dose prescribed (ml/day or g/day). For both concentration and daily dose, some CBMPs were prescribed within a range, for example, 200–220 mg/g for concentration and 0.1–2 g/day. Where this is present, the halfway value was taken for each, that is, 210 mg/g and 0.15 g/day in the above example.

### 2.4 Outcomes of interest

The primary outcomes of interest were changes in PROMs from baseline to follow‐up at 1, 3, and 6 months. The baseline PROM was recorded electronically prior to the initial prescription for CBMP was written. Follow‐up time periods were determined according to the date of the first CBMP prescription. Follow‐up periods were compared to baseline to allow comparison to health status prior to initiation of CBMPs.

AEs were either self‐reported by patients remotely contemporaneously, at 1, 3, and 6 months, and every 6 months thereafter, or were recorded by their clinician during a routine visit. AEs were categorized using the Common Terminology Criteria for Adverse Events v4.0 (Williams et al., 2003).

Concurrent medications taken by patients were recorded according to the SNOMED CT code, with changes in medications recorded throughout treatment by patients, and supplemented by clinicians if unreported between clinical encounters.

### 2.5 Single‐Item Sleep‐Quality Scale

The Single‐Item Sleep‐Quality Scale (SQS) is a sleep‐quality assessment tool which utilizes a numerical rating scale. Participants rate their sleep quality subjectively on a scale of 0–10 where “0” and “10” are equivalent to “terrible” and “excellent” sleep quality, respectively (Hurst & Bolton, 2004; Snyder et al., 2018). An increase equal to or more than 2.6 from baseline is deemed clinically significant (Snyder et al., 2018). The SQS has good convergent construct validity when investigated against the Pittsburgh Sleep‐Quality Index (correlation coefficient: −0.72) and moderate test–retest reliability in individuals with insomnia (intraclass correlation = 0.62) (Snyder et al., 2018).

### 2.6 Generalized Anxiety Disorder‐7

The Generalized Anxiety Disorder‐7 (GAD‐7) scale is a 7‐item rating system, which is utilized in the diagnosis and assessment of the severity of GAD. It has been shown to have good internal consistency (Cronbach α = 0.92) and test–retest reliability (intraclass correlation = 0.83) (Spitzer et al., 2006). Participants are asked how often they have been bothered by various symptoms of anxiety over the last 2 weeks (“0” = “not at all” to “3” = “nearly every day”). The scores are totaled to generate a score from 0 to 21. Mild, moderate, and severe anxiety is defined as ≥5, ≥10, and ≥15, respectively (Spitzer et al., 2006).

### 2.7 EQ‐5D‐5L

The EQ‐5D‐5L is a population‐based tool for the assessment of HRQoL across five domains, (mobility, self‐care, usual activities, pain or discomfort, anxiety, or depression) with five levels of severity (“1” = “no problems” to “5” = “extreme problems”). From these, a 5‐digit code is generated, then mapped to UK‐specific EQ‐5D‐5L Index values as described by van Hout et al., the preferred methodology of measuring HRQoL by NICE (NICE, 2019; Van Hout et al., 2012). An EQ‐5D‐5L Index score of 1 represents full health, whereas a score of <0 represents a health status that is worse than death. Test–retest validity of the index score has been demonstrated to be stable (≥0.70) over time across multiple settings, while internal consistency is not applicable due to being a preference‐based measure (Brazier & Deverill, 1999; Feng et al., 2021).

### 2.8 Patient global impression of change

The patient global impression of change (PGIC) is a 7‐point scale that has been validated as a gold standard of clinically significant change in health status in response to treatment (Hurst & Bolton, 2004). Patients rate their change on a numerical rating scale from 1 to 7 (“7” = “very much improved”; “6” = “much improved”; “5” = “minimally improved”; “4” = “no change”; “3” = “minimally worse”; “2” = “much worse”; “1” = “very much worse”) (Ferguson & Scheman, 2009). Test–retest validity across multiple conditions has demonstrated an intraclass coefficient between 0.53 and 0.85 (Eremenco et al., 2022).

### 2.9 Statistical analysis

Demographic variables, comorbidities, drug and alcohol use, medication data and AEs were analyzed using descriptive statistics. The normality of the distributions of each PROM data set was determined utilizing the Shapiro–Wilk test. Unless otherwise stated, parametric continuous data are presented as mean (± standard deviation), and nonparametric continuous data are presented as median (interquartile range; IQR). Statistical analysis was performed using paired *t*‐tests or Wilcoxon rank‐sum test if data were parametric or nonparametric, respectively. Missing data were handled using pairwise deletion. Effect size (*r*) was calculated for the Wilcoxon rank‐sum test as the Z‐value divided by the square root of the number of participants (*n*). The effect size was classified as large (*r* = –0.5), medium (*r* = –0.3), and small (*r* = –0.1). Statistical significance was defined as *p* < .050.

## 3 RESULTS

### 3.1 Patient data

There were 3546 patients enrolled on UKMCR on the date of data extraction (February 15, 2022). Of these, 443 were excluded for not having completed PROMs at baseline. A further 270 for a treatment duration of less than 1 month. Of the remaining 2833 patients, 61 had a primary indication for treatment with CBMPs of insomnia. In total, 50, 40, and 27 patients had PROMs recorded at 1, 3, and 6 months, respectively.

### 3.2 Baseline demographics

Demographic data about participants included in the study were analyzed (Table 1). The mean age was 41.3 (±13.0) years and the mean BMI was 26.8 (±4.9) kg/m^2^. Forty‐four patients (72.1%) were male, and 17 patients (27.9%) were female. Regarding occupation, the categories with the highest number recorded were “unemployed” and “professional” with nine patients each (14.8%). The median Charlson Comorbidity Index Score was 0.0 (0.0–0.0).

**TABLE 1:** Baseline data on patient demographics and medical history (n = 61)

**Demographic details**	**n (%)/mean ± SD**
Sex	
Male	44 (72.1%)
Female	17 (27.9%)
Age (years)	41.3 ± 13.0
Occupation	
Employed	44 (72.1%)
Clerical support workers	1 (1.6%)
Craft and related trades workers	4 (6.6%)
Elementary occupations	3 (4.9%)
Managers	4 (6.6%)
Other occupations	8 (13.1%)
Professional	9 (14.8%)
Service and sales workers	5 (8.2%)
Skilled agricultural, forestry and fishery workers	2 (3.3%)
Technicians and associate professionals	8 (13.1%)
Unemployed	9 (14.8%)
Unspecified	8 (13.1%)
BMI (kg/m^2^)	26.8 ± 4.9

Medical history	n (%)/median (IQR)
Charlson Co‐morbidity Index Score	0.0 (0.0‐0.0)
Comorbidities	
Hypertension	2 (3.3%)
Depression/anxiety	26 (42.6%)
Arthritis	1 (1.64%)
Epilepsy	2 (3.29%)
Venous thromboembolism	0 (0%)
Endocrine thyroid dysfunction	2 (3.29%)
Smoking status	
Never smoked	16 (26.2%)
Ex‐smoker	26 (42.6%)
Current smoker	19 (31.1%)
Smoking pack years (current or ex‐smokers)	9.0 (2.0 ‐ 20.0)
Weekly units of alcohol consumption	4.0 (0.0 ‐ 12.0)
Recreational cannabis use	
Never used	15 (24.6%)
Ex‐user	15 (24.6%)
Current user	31 (50.8%)
Cannabis gram years (current or ex‐users)	5.5 (1.0 ‐ 14.5)
Insomnia medication at baseline	
Diazepam	5 (8.2%)
Zopiclone	13 (21.3%)
Zolpidem	2 (3.3%)
Not currently on medication for insomnia	41 (67.2%)

Forty‐five patients (73.7%) were either current or ex‐smokers with a median pack‐year history of 9.0 (2.0–20.0). The median weekly alcohol consumption was 4.0 (0.0–12.0) units. Forty‐six patients (75.4%) were either current or ex‐users of recreational cannabis with a median exposure of 5.5 (1.0–14.5) cannabis gram years.

Fifteen patients (24.6%) were on Z‐drugs and five patients (8.2%) were prescribed diazepam at the time of data extraction, while 41 patients (67.2%) were not present on any medication for insomnia.

All 61 patients had a primary indication of insomnia for treatment with CBMPs. Secondary and tertiary indications for treatment with CBMPs are detailed in Table 2.

**TABLE 2:** Primary, secondary, and tertiary indications for cannabis‐based medicinal products of study participants (*n* = 61)

Indication	Primary *n* (%)	Secondary *n* (%)	Tertiary *n* (%)
Insomnia	61 (100%)	–	–
Anxiety	–	17 (27.9%)	2 (3.3%)
Depression	–	3 (4.9%)	5 (8.2%)
Autism spectrum disorder	–	0 (0.0%)	1 (1.6%)
Chronic pain	–	5 (8.2%)	0 (0.0%)
Epilepsy adult	–	0 (0.0%)	1 (1.6%)
Headache	–	1 (1.6%)	0 (0.0%)
Migraine	–	5 (8.2%)	0 (0.0%)
Posttraumatic stress disorder	–	2 (3.3%)	0 (0.0%)

### 3.3 Cannabis‐based medicinal products

Data on prescribed CBMPs was available for 57 (93.4%) individuals (Table 3). Dry flower preparations alone were prescribed to 24 patients (42.1%), oral or sublingual oils were prescribed to 16 patients (28.1%) and 17 patients (29.8%) were prescribed both. The most prescribed vaporized dry flower preparation was Adven® 20% Δ^9^‐THC EMT2 hybrid flos (Curaleaf International, Guernsey, UK) and the most prescribed oil preparation was Adven® 20 mg/mL Δ^9^‐THC full spectrum hybrid/indica oil (Curaleaf International, Guernsey, UK). The median daily Δ^9^‐THC dose for the entire cohort was 120.0 mg/24 h (23.2–195.0 mg/24 h) and the median daily initial CBD dose was 5.0 mg/24 h (0.0–20.0 mg/24 h).

**TABLE 3:** Recorded data on prescribed cannabis‐based medicinal products at point of maximum titration (*n* = 57).

Prescription information	*n* (%)/Median (IQR)
**Oils**	16 (28.1%)
CBD, mg/24 h	20.0 (0.3–28.8)
THC, mg/24 h	10.0 (10.0–20.0)
**Dried flower**	24 (42.1%)
CBD, mg/24 h	1.5 (0.0–5.0)
THC, mg/24 h	150.0 (100.0–195.0)
**Oils and dried flowers (combination)**	17 (29.8%)
CBD, mg/24 h	20.0 (0.8–32.8)
THC, mg/24 h	140.0 (118.0–217.5)

### 3.4 Patient‐reported outcome measures

PROMs are reported in Table 4 in full. SQS showed change from baseline at 1 month (*p* < .001), 3 months (*p* < .001), and 6 months (*p* < .001). GAD‐7 scores showed change from baseline at 1 month (*p* < .001), 3 months (*p* < .001), and 6 months (*p* = .003) (Table 4). Twenty‐nine (42.0%), 20 (50.0%), and 14 (51.9%) participants with complete follow‐up experienced clinically significant changes in SQS at 1, 3, and 6 months respectively. EQ5D5L showed change from baseline at 1 month (*p* = .003), 3 months (*p* = .002) and 6 months (*p* = .024). The median PGIC value remained constant at 1 month (*n* = 49; 6.00; 5.00–6.00), 3 months (*n* = 39; 6.00; 5.00–7.00), and 6 months (*n* = 26; 6.00; 5.00–6.75). There were no statistically significant differences between reported PROMs at 3 and 6 months compared to the preceding follow‐up period (*p* > .050).

**TABLE 4:** Median (IQR) baseline and follow‐up scores for GAD‐7, SQS, and EQ‐5D‐5L at 1, 3, and 6 months.

		*n*	Baseline score	Follow‐up score	*p* Value	*t*‐Test statistic	*Z*‐score	Effect size (*r*)
**SQS**	1 month	50	3.00 (2.00–5.00)	6.00 (4.00–8.00)	<.001	642.00	–4.88	–0.69
	3 months	40	3.00 (2.00–5.00)	6.00 (5.00–8.00)	<.001	644.00	–4.90	–0.77
	6 months	27	3.00 (2.00–5.00)	6.00 (5.00–8.00)	<.001	292.00	–4.08	–0.79
**GAD‐7**	1 month	50	7.00 (2.25–11.00)	3.00 (0.25–6.00)	<.001	841.00	–4.46	–0.63
	3 months	40	7.00 (2.00–10.25)	1.50 (0.00–6.00)	<.001	561.00	–4.51	–0.71
	6 months	27	6.50 (2.00–9.00)	2.00 (1.00–4.00)	.003	290.00	–3.45	–0.66
**EQ‐5D‐5L Mobility**	1 month	50	1.00 (1.00–1.00)	1.00 (1.00–1.00)	.380	9.00	–0.88	–0.12
	3 months	40	1.00 (1.00–1.00)	1.00 (1.00–1.00)	.564	4.00	–0.58	–0.09
	6 months	27	1.00 (1.00–1.00)	1.00 (1.00–1.00)	.655	2.00	–0.45	–0.09
**EQ‐5D‐5L Self‐Care**	1 month	50	1.00 (1.00–1.00)	1.00 (1.00–1.00)	.561	13.50	–0.65	–0.09
	3 months	40	1.00 (1.00–1.00)	1.00 (1.00–1.00)	.414	14.00	–0.82	–0.13
	6 months	27	1.00 (1.00–1.00)	1.00 (1.00–1.00)	.317	1.00	–1.00	–0.19
**EQ‐5D‐5L Usual Activities**	1 month	50	1.00 (1.00–2.00)	1.00 (1.00–1.75)	.007	129.00	–2.71	–0.38
	3 months	40	1.00 (1.00–2.00)	1.00 (1.00–2.00)	.011	113.50	–2.56	–0.40
	6 months	27	1.00 (1.00–2.00)	1.00 (1.00–2.00)	.083	36.00	–1.73	–0.33
**EQ‐5D‐5L Pain and Discomfort**	1 month	50	2.00 (1.00–3.00)	2.00 (1.00–2.00)	.012	182.50	–2.52	–0.36
	3 months	40	2.00 (1.00–3.00)	2.00 (1.00–2.00)	.003	163.00	–3.00	–0.47
	6 months	27	2.00 (1.00–3.00)	1.00 (1.00–2.00)	.061	53.50	–1.87	–0.36
**EQ‐5D‐5L Anxiety and Depression**	1 month	50	2.00 (1.00–3.00)	2.00 (1.00–2.75)	.001	202.50	–3.23	–0.46
	3 months	40	2.00 (1.00–3.00)	1.00 (1.00–2.00)	.010	195.00	–2.95	–0.47
	6 months	27	2.00 (1.00–3.00)	2.00 (1.00–2.00)	.097	74.50	–2.14	–0.41
**EQ‐5D‐5L Index value**	1 month	50	0.76 (0.68–0.90)	0.83 (0.75–1.00)	.003	422.00	–2.96	–0.42
	3 months	40	0.77 (0.69–0.93)	0.84 (0.76–1.00)	.002	361.50	–3.11	–0.49
	6 months	27	0.77 (0.72–0.88)	0.85 (0.74–1.00)	.024	151.00	–2.25	–0.43

*Note*: *n* and the baseline scores differ according to the period due incomplete follow‐up for the corresponding study time point.

### 3.5 Adverse events

Twenty‐eight (45.9%) adverse effects were recorded by 8 (13.1%) participants. The most common AEs were insomnia (*n* = 5; 17.9%), dry mouth (*n* = 3; 10.7%), and dizziness (*n* = 3; 10.7%) (Table 5). There were no life‐threatening/disabling AEs.

**TABLE 5:** Adverse events recorded by participants (*n* = 61).

Adverse event	Mild	Moderate	Severe	Total (%)
Insomnia	0	3	2	5 (17.9%)
Dry mouth	2	1	0	3 (10.7%)
Fatigue	2	0	0	2 (7.1%)
Dizziness	2	1	0	3 (10.7%)
Somnolence	0	1	0	1 (3.6%)
Lethargy	2	0	0	2 (7.1%)
Tremor	2	0	0	2 (7.1%)
Headache	2	0	0	2 (7.1%)
Vertigo	1	1	0	2 (7.1%)
Constipation	1	0	0	1 (3.6%)
Delirium	1	0	0	1 (3.6%)
Nausea	1	0	0	1 (3.6%)
Concentration impairment	1	0	0	1 (3.6%)
Amnesia	1	0	0	1 (3.6%)
Heart palpitations	0	1	0	1 (3.6%)
**Total (%)**	**18 (29.5%)**	**8 (13.1%)**	**2 (3.3%)**	**28** **(45.9%)**

## 4 DISCUSSION

This case series investigated an insomnia patient cohort treated with CBMPs. There was an improvement in subjective sleep quality as evidenced by the large effect size seen in change in SQS score. More than 40% of participants who completed each PROM round reported clinically significant improvement in their sleep quality at each time period. These results show that initiation of CBMP therapy was associated with improvements in those patients who had previously failed to respond to currently licensed treatments for insomnia. Improvements in GAD‐7, SQS, and EQ‐5D‐5L Index values at 1, 3, and 6 months (*p* < .050) were witnessed after CBMP commencement. EQ‐5D‐5L subscores for usual activity, pain and discomfort, and anxiety and depression also improved at 1 and 3 months (*p* < .050). The incidence of AEs was 28 (45.9%), and most were either mild or moderate. A case series study design, however, limits the extent to which a causal relationship can be determined irrespective of statically significant changes in the observed measures.

A previous case series of 72 patients found sleep scores to improve for 66.7% of the patients within the first month, however the score fluctuated over time (Shannon et al., 2019). While the present study found a similar improvement in sleep‐quality scores after the first month of CBMP initiation, the magnitude of change was largely consistent over time. The difference in response may be secondary to the differences in active treatment used. While the present study utilized CBMPs including Δ9‐THC, the Shannon et al. (2019) study only observed treatment response to CBD isolate therapy. Other observational studies and randomized controlled trials, internationally, have also found similar results across a heterogeneous selection of CBMPs (Aminilari et al., 2022; Lavender et al., 2022; Ried et al., 2023). A 2017 literature review found that Δ9‐THC may be particularly beneficial in reducing sleep latency; however, the long‐term effects of tolerance to these effects are not known (Babson et al., 2017). This effect on sleep latency provides a possible explanation for the clinically and statistically significant improvements in SQS, although the short objective time frame of 6 months means that future analysis will be required to identify any tolerance to these effects.

Participants reported improvements in anxiety symptoms at each follow‐up. This is consistent with similar findings from other analyses of the UKMCR considering those with a diagnosed GAD, and those with other conditions (Ergisi et al., 2022; Harris et al., 2022). There is a paucity of RCT data on the efficacy of CBMPs in anxiety disorders beyond social anxiety disorder; however, there is clear preclinical evidence of the role of cannabinoids on emotional regulation, including fear and anxiety (Black et al., 2019; Ebbert et al., 2018). Although the mechanisms are incompletely understood, anxiety and insomnia are believed to have a bidirectional effect on one another (Blake et al., 2018; Jansson & Linton, 2006). Therefore, CBMPs may provide auxiliary benefits beyond modulation of the sleep‐wake cycle in insomnia. However, it was not possible from the present analysis, due to sample size, to conduct a subgroup analysis according to whether the presence of state anxiety affects the magnitude of response to CBMPs when prescribed.

It has previously been shown that HRQoL is severely impaired in those with insomnia (Ishak et al., 2012). Limited evaluation of the use of hypnotics, such as benzodiazepines and Z‐drugs, in insomnia has failed to show any change in outcomes for HRQoL (Scalo et al., 2015). In other settings, high‐dose benzodiazepines have shown deleterious effects on HRQoL (Cheng et al., 2020; Tamburin et al., 2017). The associated improvement in HRQoL in this study is consistent with changes previously identified in a Canadian cohort who received medical cannabis for insomnia (Vaillancourt et al., 2022). However, the present study builds upon this prior work by utilizing a validated measure, the EQ‐5D‐5L, to assess HRQoL. Direct comparison between CBMPs and currently utilized therapies or placebo through RCTs will ultimately be necessary, however, to determine its true effects on HRQoL.

The present study had an AE incidence of 28 (45.9%), with 18 (29.5%) being mild, 8 (13.1%) being moderate, and 2 (3.3%) being severe. The reported literature on AEs following the administration of CBMPs is heterogeneous. A 2008 systematic review found the overall AE incidence of CBMPs from the 23 RCTs was 4779 (247.4%), 96.6% of which were nonfatal AEs (Wang et al., 2008). A 2‐week RCT of a CBMP for insomnia found similar adverse effects to the present study from a cohort of 24 patients with chronic insomnia but with an addition of sensorineural adverse effects, such as “feeling abnormal,” ataxia, and auditory and visual hallucinations (Walsh et al., 2021).

The present study is subject to several limitations. As a case series, without control or randomization, it is not possible to conclude that CBMPs were solely responsible for the changes in sleep‐specific outcomes and general HRQoL. In addition to this, the study is subject to a sampling bias, with many participants reporting prior cannabis use at study baseline. While the present study has high external validity, significant clinical heterogeneity remains. Due to the present size of the insomnia cohort within the UKMCR at the time of this analysis there was insufficient data to conduct specific subanalyses according to specific patient or product characteristics. In the future as the size of the UK Medial Cannabis Registry continues to grow assessments of the effects seen in individuals prescribed specific CBMPs or in those naïve to cannabis should be explored. Moreover, collection and measurement of outcomes may be subject to limitations. The reporting PROMs are subject to recall bias. The collection of CBMP data avoids this through using data extracted from prescriptions, rather than patient‐reported data. However, this may fail to account for noncompliance with the CBMP prescription. Finally, as the inclusion criteria for this present study was limited to those included for 1 month or longer in the UKMCR, future evaluations should seek to set this at 6 months or more to enable more robust treatment of missing data.

## 5 CONCLUSIONS

This novel case series assessed patients suffering from insomnia prescribed CBMPs for up to 6 months, showing an associated improvement in self‐reported sleep quality, generalized anxiety and general HRQoL. While approximately 40% or more individuals experienced a clinically significant improvement in sleep quality, it is important to recognize that these findings must be interpreted within the limitations of the study design. Ultimately, RCTs will be necessary to determine the true efficacy of CBMPs for insomnia. Moreover, longer‐term analyses will be required to determine whether there is an effect of tolerance on CBMP efficacy in insomnia. However, the results do suggest that CBMPs are largely well tolerated by most individuals within 6 months of follow‐up.

## AUTHOR CONTRIBUTIONS

Kavyesh Vivek, Zekiye Karagozlu, Simon Erridge, Carl Holvey, Ross Coomber, James J. Rucker, Mark W. Weatherall, and Mikael H. Sodergren contributed to the study conception and design. Kavyesh Vivek, Zekiye Karagozlu, Simon Erridge, Carl Holvey, Ross Coomber, James J. Rucker, and Mark W. Weatherall contributed to the acquisition of data. Kavyesh Vivek, Zekiye Karagozlu, Simon Erridge, Mark W. Weatherall, and Mikael H. Sodergren contributed to the analysis and interpretation of data. Kavyesh Vivek, Zekiye Karagozlu, and Mikael H. Sodergren contributed to the drafting of the manuscript. Kavyesh Vivek, Zekiye Karagozlu, Simon Erridge, Carl Holvey, Ross Coomber, James J. Rucker, Mark W. Weatherall, and Mikael H. Sodergren contributed to critical revision. All authors agreed to be accountable for all aspects of the work and approved the final manuscript.

## CONFLICT OF INTEREST STATEMENT

SE, CH, RC, JJR, MWW, and MHS are the founding clinicians of Curaleaf Clinic, which is the first clinic registered with the CQC to evaluate patients for medical cannabis in England. The policy of Curaleaf Clinic is to disclose the results of all research studies, irrespective of the reported outcomes.

## FUNDING INFORMATION

There was no external or commercial funding associated with this paper.

### PREVIOUS PUBLICATION

This original paper has not been previously published or simultaneously submitted for publication elsewhere. The data have been presented as a poster at the 2022 International Cannabinoid Research Society Conference.

### PRINCIPAL INVESTIGATOR

The authors confirm that the PI for this paper is Mikael H Sodergren and that he had direct clinical responsibility for patients.

### PATIENT CONSENT

All participants completed written, informed consent before enrolment in the registry.

### PEER REVIEW

The peer review history for this article is available at https://publons.com/publon/10.1002/brb3.3410.

## Data Availability

Data were derived from the UK Medical Cannabis Registry (ukmedicalcannabisregistry.com) and restrictions apply. Please contact the corresponding author directly for further details.
